# Divergent effects of oxytocin on “mind-reading” in healthy males

**DOI:** 10.3758/s13415-021-00936-3

**Published:** 2021-09-13

**Authors:** Ana Macchia, Paul Theo Zebhauser, Stephanie Salcedo, Bethany Burum, Edward Gold, Miguel Alonso-Alonso, Alvaro Pascual-Leone, Daniel Gilbert, Anna-Katharine Brem

**Affiliations:** 1 Max Planck Institute of Psychiatry, Munich, Germany; 2 Clinic for Psychiatry/Psychotherapy III, Ulm University, Ulm, Germany; 3 Department of Neurology, Klinikum Rechts der Isar der Technischen Universität München, Munich, Germany; 4 Department of Psychiatry and Psychotherapy, Klinikum Rechts der Isar der Technischen Universität, Munich, Germany; 5 Department of Psychology, Harvard University, Cambridge, MA USA; 6 Berenson-Allen Center for Noninvasive Brain Stimulation, Division of Interventional Cognitive Neurology, Department of Neurology, Beth Israel Deaconess Medical Center, Harvard Medical School, Boston, MA USA; 7 Guttmann Brain Health Institute, Institut Guttmann, Universitat Autonoma Barcelona, Barcelona, Spain; 8 Department of Neurology, Harvard Medical School, Boston, MA USA; 9 Hinda and Arthur Marcus Institute for Aging Research and Center for Memory Health, Hebrew SeniorLife, Boston, MA USA; 10 University Hospital of Old Age Psychiatry, Murtenstrasse 21, 3008 Bern, Switzerland; 11 Department of Neuropsychology, Lucerne Psychiatry, Lucerne, Switzerland

**Keywords:** Oxytocin, Theory of mind, RMET, Social cognition

## Abstract

The neuropeptide oxytocin (OT) has been associated with a broad range of human behaviors, particularly in the domain of social cognition, and is being discussed to play a role in a range of psychiatric disorders. Studies using the Reading The Mind In The Eyes Test (RMET) to investigate the role of OT in mental state recognition reported inconsistent outcomes. The present study applied a randomized, double-blind, cross-over design, and included measures of serum OT. Twenty healthy males received intranasal placebo or OT (24 IU) before performing the RMET. Frequentist and Bayesian analyses showed that contrary to previous studies (Domes et al., 2007; Radke & de Bruijn, 2015), individuals performed worse in the OT condition compared to the placebo condition (*p* = 0.023, Cohen’s *d* = 0.55, 95% confidence interval [CI] [0.08, 1.02], BF_10_ = 6.93). OT effects did not depend on item characteristics (difficulty, valence, intensity, sex) of the RMET. Furthermore, OT serum levels did not change after intranasal OT administration. Given that similar study designs lead to heterogeneous outcomes, our results highlight the complexity of OT effects and support evidence that OT might even interfere with social cognitive abilities. However, the Bayesian analysis approach shows that there is only moderate evidence that OT influences mind-reading, highlighting the need for larger-scale studies considering the discussed aspects that might have led to divergent study results.

**Supplementary Information:**

The online version contains supplementary material available at 10.3758/s13415-021-00936-3.

“Mind-reading” often is mentioned in the context of science fiction and referred to as a supernatural or telepathic ability to quickly detect another's thoughts or feelings (Barrett et al., 1882). In reality, humans lack such direct access to other minds. Humans use hints to be able to "read" or "interpret" the internal states of others, which provides the basis for appropriate behavioral responses. Impairments in this core component of social cognition are prominent in a range of psychiatric disorders (Luyten & Fonagy, 2015). The ability to simulate the internal state of others is commonly operationalized using the "Reading the Mind in the Eyes Test" (RMET), a test that probes recognition of complex emotional expressions (Baron-Cohen et al., 2001). Low-level perceptual mechanisms, as well as emotion recognition and complex mental state deduction, requiring theory of mind (ToM), have been suggested to be involved in the execution of the RMET (Mitchell & Phillips, 2015).

“Mind-reading” is only one in a range of human behaviors that have been associated with the neuropeptide oxytocin (OT), which further includes loving, trusting, caregiving, parenting, sexual behavior, and aggression (de Jong & Neumann, 2017; Declerck et al., 2020; Hurlemann et al., 2010). Recent findings indeed point toward a diverse portfolio of effects and a more general role of OT in social behavior (Harari-Dahan & Bernstein, 2014; Shamay-Tsoory & Abu-Akel, 2016). By operating as a hormone and neurotransmitter, oxytocin affects the brain and has various effects on peripheral organs. In the central nervous system, OT receptors can be mainly found in limbic areas and the basal forebrain of the human brain (Baribeau & Anagnostou, 2015), emphasizing the role of OT in emotional processing and regulation (Tully et al., 2018).

In recent years, a plethora of studies have investigated effects of OT administration in healthy subjects and different psychiatric conditions on behavior, including ToM and emotional processing. Mixed results have been reported in depression (MacDonald et al., 2013), schizophrenia (Brambilla et al., 2016; Feifel et al., 2010), autism (Parker et al., 2017), eating disorders (Leppanen et al., 2017a), and addiction (Woolley et al., 2016). A meta-analysis that summarized the effect of intranasal OT on ToM in 14 studies concluded that neither healthy samples nor clinical populations benefited from OT when interpreting complex emotions (Leppanen et al., 2017b). Problematically, there have been few replication attempts in the field of intranasal OT research, and the few that have been implemented were largely unsuccessful (Declerck et al., 2020; Mierop et al., 2020). There have been various considerations to improve the precision of OT research. By including previous knowledge about a research field and providing evidence for or against a null hypothesis, Bayesian data analysis might be one of such tools to enhance the reproducibility of OT research (Winterton et al., 2021).

In a pioneering study, Domes et al. reported that OT improves the ability to decipher mental states using the RMET (Domes et al., 2007). The authors investigated healthy males in a double-blind, placebo-controlled, within-subject study, 20 out of 30 individuals performed better on the RMET after intranasal OT administration, with a mean increase of approximately 3% of correct responses. These findings were corroborated in a between-subject design, but in this study enhancing effects of OT became only apparent in participants with lower baseline empathy and in more difficult items (Feeser et al., 2015). Subsequently, Radke and de Bruijn (2015) undertook a replication and extension of Domes’ et al. (2007) study and found no effect on “mind-reading,” even when considering item difficulty and carefully evaluating several other parameters, such as valence, intensity, and sex of the presented facial expressions. Nevertheless, the study indicated that individuals with lower baseline empathy showed larger improvement in RMET-performance after OT administration.

In sum, previous studies investigating the effect of OT on “mind-reading” in healthy males indicated that (1) effects of intranasally administered OT on “mind-reading” are inconsistent, (2) if OT has any effect on “mind-reading” it might be more pronounced for emotions that are difficult to interpret, and that (3) not all participants benefit from OT, but maybe especially those with lower baseline empathic abilities.

The primary goal of the present study was to reexamine the effect of OT on “mind-reading.” The present study employed a similar study design as previous studies by Domes et al. (2007) and Radke and de Bruijn (2015). To quantify evidence for the effect of OT on “mind-reading,” we additionally provide Bayesian statistics for ours and previous studies (Domes et al., 2007; Radke and de Bruijn, 2015). We investigated the effect of OT versus placebo (PLC) performance on the RMET in young healthy men, the association between empathic abilities and OT effects, and extended the design to include peripheral serum measures of OT. We included OT blood measures to detect changes in OT blood levels after intranasal administration and examine possible associations with behavioral changes.

## Methods

### Sample

A total of 24 healthy males (*M* = 22.85 years, *SD* = 3.5) were randomly assigned to receive 24 IU of intranasal OT (Syntocinon, Novartis, Basel, Switzerland) by taking three puffs per nostril (4 IU per puff) or PLC (saline solution that contained all ingredients except the neuropeptide) on two study visits that were scheduled one week apart. Further tasks unrelated to our main research question were administered after the end of the second visit and on a third visit (results will be reported separately). Participants were recruited through advertisements on university campuses and were compensated with $50 per session. Visits took place at the Harvard Thorndike Clinical Center at Beth Israel Deaconess Medical Center, Boston, MA. Exclusion criteria for participation were any current or past history of psychiatric illness, unstable medical condition, smoking, nasal pathologies, and current abuse or dependence in the past 6 months of drugs or alcohol. Participants received instructions to abstain from food for 9 hours, from drinks (other than water) for 2 hours, and from alcohol and caffeine for 24 hours before study visits. All study visits were scheduled to start at 9 am. Before each study visit participants were reminded of intake limitations via phone calls.

The local Institutional Review Boards of Beth Israel Deaconess Medical Center and Harvard University approved the study. All participants gave written informed consent before the study onset according to the Declaration of Helsinki.

### Instruments

The RMET measures how well people are able to “read” facial expressions by subtle social cues. It consists of photos of people’s eye regions depicting different mental states. Participants have to choose the most appropriate mental state from a selection of four possible answers as quickly as possible. The computer-based revised English version (Baron-Cohen et al., 2001) consists of 36 items and one practice trial. Stimuli were always presented in the same order and presentation time was not time-limited. RMET items can be classified in terms of the following variables: task difficulty (easy vs. difficult), intensity (i.e., strength of the emotional state; low vs. high), valence (i.e., pleasantness of the emotional expression; positive vs. negative), and sex (female vs. male eye regions). To investigate mood state, the English version of the Multidimensional Mood Questionnaire (MDMQ) (Steyer et al., 1994) was administered after the RMET in both experimental conditions (OT, PLC). It contains 30 items with bipolar dimensions assessing good and bad mood, alertness and tiredness, and calm and nervous states. Online questionnaires assessed self-reported trait empathy with the Interpersonal Reactivity Index (IRI) (Davis, 1980) and the Empathy Quotient (EQ) (Baron-Cohen & Wheelwright, 2004). The IRI is a multidimensional tool to measure empathy consisting of four subscales: a perspective taking scale, an empathic concern scale, a personal distress scale, and a fantasy scale (Davis, 1980). The EQ is a 60-item questionnaire with a maximum score of 80 (Baron-Cohen & Wheelwright, 2004). Impulsivity was measured with the Barratt Impulsivity Scale (BIS) (Patton et al., 1995). Thirty items are categorized into six first-order factors, which build the second-order factors "attentional impulsiveness," "motor impulsiveness," and "nonplanning impulsiveness." Participants, study procedures, and psychological tasks of our study and previous studies are summarized in Table 1.

**Table 1:** Characteristics of the current sample and study design compared to previous studies

	Current	Radke & de Bruijn (2015)	Domes et al. (2007)
Sample (final analysis)	20 healthy males	24 healthy males	30 healthy males
Mean age, yr (SD)	22.85 (±3.5)	21.5 (±1.9)	25.3 (±2.2)
OT dose and visit interval	24IU, 1 week (administration order randomized)	24IU, 2 weeks (administration order randomized)	24IU, 1 week administration order balanced)
RMET start (min after OT administration)	45	50 or 65 (counterbalanced)	45
Activities during waiting period (min after administration)	Watching movie with nature scenes	Answering questionnaires; free to read/study/other occupations	Reading news magazines
Country	US	Netherlands	Germany
Exclusion criteria	Psychiatric disease; unstable medical condition; smoking; nasal pathologies; drug/alcohol abuse (current abuse or in the past 6 months)	Age <18 or >30 yr; neurological/ endocrine disease; medication use; smoking >5 cigarettes/day; participation in pharmacological study within 2 months before inclusion; sickness on test day (fever, cold, allergic rhinitis)	Medical/psychiatric disease; medication use; smoking; substance abuse
Abstinence	Food: 9 h Drink (except water): 2 h Alcohol/caffeine: 24 h Nicotine: exclusion criteria	Food: 2 h Drink (except water): 2 h Alcohol/caffeine: 24 h Nicotine: 24 h	Food: 2 h Drink (except water): 2 h Alcohol/caffeine: 2 h Nicotine: 2 h
Recruitment	Advertisement on university campus and online postings	Advertisement on university campus and online recruitment system	Advertisement on university campus
Questionnaires	EQ IRI MDMQ BIS	EQ IRI VAS STAXI SAS	MDMQ
Incentive	Financial compensation (50$)	Financial compensation (50€)	None

BIS, Barratt Impulsiveness Scale; EQ, Empathy Quotient; IRI, Interpersonal Reactivity Index; MDMQ, Multidimensional Mood Questionnaire; RMET, Reading the Mind in the Eyes Test; STAXI, State-Trait Anxiety Inventory; SAS, Social Anxiety Scale; VAS, visual analogue scale for nonspecific effects of OT.

## Design

**Fig. 1:**
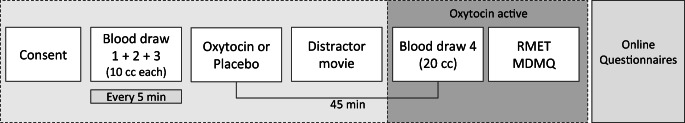
Detailed study procedure for each visit. After giving written, informed consent, the IV was inserted and three baseline blood samples (pre) were collected, spaced 5 min apart. Participants randomly received intranasal PLC or OT. While waiting 45 min for the OT effect to fully emerge (Spengler et al., 2017), individuals watched a distractor movie showing nature scenes. During the waiting period, subjects were left alone and were not allowed to use a mobile phone to minimize human interaction. After the waiting period, a fourth blood sample (post) was drawn. Then, the RMET and the Multidimensional Mood Questionnaire (MDMQ) (Steyer et al., 1994) were performed. Other questionnaires were administered via an online tool. Both the OT and the PLC visit followed the same procedure.

Figure 1 shows the detailed study procedure. The study followed a randomized, double-blind, cross-over design. Participants answered questionnaires online through Research Electronic Data Capture (REDCap) (Harris et al., 2009) after the second visit. Possible adverse events related to OT administration (irritation of the nasal mucosa, nausea, headache, change in mood, allergic dermatitis, and other events) were assessed using a questionnaire before and after each session. No significant adverse events occurred. Details are reported in the supplemental material. A research nurse instructed and supervised individuals on the administration of OT. OT, as well as the PLC, were acquired through the same company (Victoria Pharmacy, Zurich, Switzerland) and presented in identical containers. Using appropriate procedures, subjects are not able to detect differences between OT and PLC (MacDonald et al., 2011). After assessing vital signs (oral temperature, blood pressure, heart rate), the nurse inserted the intravenous catheter (IV) and a distractor movie showing nature scenes started. A total of 50 cc of blood was drawn before and after intranasal administration of either OT or placebo. Baseline blood draws began 15 minutes after inserting the IV. In order to reduce baseline variability, the baseline draw was divided into three draws of 10 cc spaced 5 min apart providing us with a mean baseline OT level (Lefevre et al., 2017). Immediately after the baseline blood draw, participants received OT/PLC and after a 45-min break, the fourth blood draw (20 cc) took place before starting the tasks. Human interaction was kept to a minimum during the entire session. Blood draws were performed while subjects were reclining in a bed. Serum OT levels were measured following extraction using an enzyme immunoassay kit from Assay Designs (Ann Arbor, MI). The intra-assay coefficient of variation (CV) was 8.7-12.4% and the inter-assay CV was 5.2-14.5%. The sensitivity was 7.0 pg/ml.

## Statistical analyses

Data were analyzed using frequentist and Bayesian analysis methods. Frequentist data analysis was performed with RStudio (R Version 3.5.1) and reanalyzed independently using IBM SPSS Statistics (Version 23.0) by two different authors. Bayesian data analysis was conducted with JASP (JASP Team, 2020). Normality was examined with the Shapiro-Wilk test (Tables S1-S3 in the supplemental material) and visual distributions. We used additional non-parametric methods for normality deviations. Results of tests dealing with non-normal data were comparable with parametric tests (details are provided in the supplemental material). The sample size decreased to 20 individuals due to drop out (*n* = 2) and technical problems (*n* = 2). One participant did not answer the online questionnaires but was included in the main analyses. Box and density plots are implemented to elucidate our data. Compared with boxplots, density plots show smoother distribution of the data. It applies kernel density estimation to retrieve the probability density function of the data. Moreover, density distributions determine the distribution shape and are useful to visualize differences between the two conditions. To determine whether OT enhances RMET-performance, we performed a two-sided paired *t*-test (OT vs. PLC). A sample of 20 participants in a within-participants design can only reliably detect (i.e., 80% power) an effect size of 0.66 or higher when performing a paired-samples *t*-test. In order to complement frequentist results, we additionally provide Bayesian data analysis. It allows making use of previous knowledge about the research field and quantifies statistical evidence for and against the alternative hypothesis (Etz & Vandekerckhove, 2018). Bayesian paired samples *t*-test was applied to all three studies (our study, Domes et al., 2007; Radke & de Bruijn, 2015) using the Summary Stats module (Ly et al., 2018). As prior distribution we used the average effect size of OT studies in healthy individuals (Cohen’s *d* = 0.28) as the central location with a Cauchy scale of 0.1, as suggested by Quintana and Williams (2018). We additionally analyzed the data using the “Oosterwijk prior” (*t*-distribution centered at 0.35, scaling factors of 0.102, 3 degrees of freedom). The “Oosterwijk prior” can be used if there is a lack of explicit prior knowledge and is considered to be a plausible prior in the field of psychology and biobehavioral science (Gronau et al., 2020; Quintana & Williams, 2018; Stefan et al., 2019).

Separate (Bayesian) repeated-measure Analyses of Variance (ANOVAs) for the potential moderating variables task difficulty (easy vs. difficult), intensity (low vs. high), valence (positive vs. negative), and sex (female vs. male eye regions), as well as the experimental condition (OT vs. PLC) were performed in order to obtain model comparisons. Item classification was obtained from Radke and de Bruijn who had followed earlier studies (Radke and de Bruijn, 2015). We compared all possible models with main and interaction effects of item classification (difficulty, valence, intensity, sex) and condition (OT, PLC) against the model providing the strongest evidence for the null hypothesis (i.e., null model). Each of the candidate models was considered equally probable and therefore assigned to the same prior probability (i.e., 1/5 = 0.2). We preserved default Cauchy priors in JASP with a width factor of 0.5 for fixed effects, 1.0 for random effects, and 0.354 for interaction effects.

Differences of the MDMQ in the OT and PLC condition were tested with (Bayesian) two-sided paired *t*-tests. (Bayesian) Pearson correlations were applied to investigate associations between empathy (EQ; IRI), impulsiveness (BIS) and the RMET in both experimental conditions (PLC, OT). Additional partial correlations (controlling for RMET performance in the PLC condition) were used to investigate the relationship between empathy and RMET performance. Pooled pre- (first, second, and third blood measure) and post- (fourth blood measure drawn 45 min later) OT serum measures were compared with a (Bayesian) paired-samples two-sided *t*-test. Additionally, (Bayesian) paired *t*-tests were used to determine whether the pre- and post-OT serum levels differed between the conditions. No predetermined hypotheses were used to guide the analysis of OT serum levels. Results of the blood level analysis are provided in the supplemental material.

## Results

### RMET

**Fig. 2:**
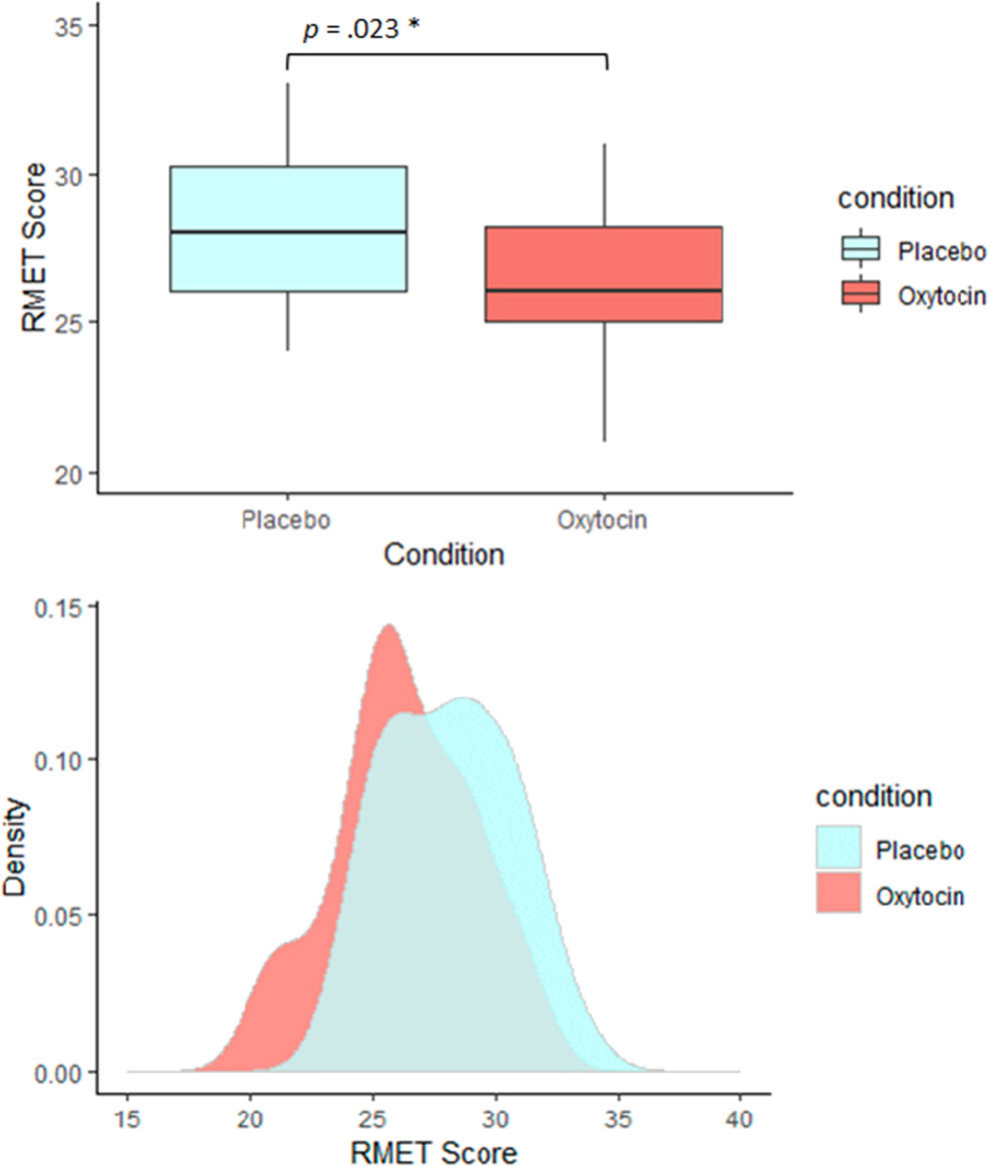
Total RMET scores in the OT and PLC conditions. Boxplots (top) and Density distributions (bottom) indicate that participants receiving OT performed worse in the RMET. Density plots show that RMET scores in the OT condition are concentrated on a lower interval compared with the PLC condition

Table 2 depicts our data in comparison to earlier studies. Unlike in previous studies, total RMET scores were significantly reduced after OT administration compared with PLC administration, indicated by a medium effect , *t*(19) = 2.47, *p* = 0.023, *d* = 0.55, 95% confidence interval [CI] [0.08, 1.02] (Table 2; Figure 2). Considering an average effect size of *d* = 0.28 of OT studies in healthy individuals (Walum et al., 2016), our data show that the alternative hypothesis (i.e., OT influences mind-reading) is 6.93 times more likely than the null model (i.e., there is no effect of OT on mind-reading). This is in line with the frequentist data analysis and can be interpreted as moderate evidence for a negative effect of OT on “mind-reading” according to the classification of Lee and Wagenmakers (2013; adjusted from Jeffreys, 1961). Domes’ et al. (2007) study shows that it is 3.29 times more plausible that there is a positive effect of OT on “mind-reading” than a null-effect, which also coincides with the frequentist analysis. In contrast, Radke’s study provides anecdotal evidence for a null-effect (BF_10_ = 1.67) and does not favor one hypothesis over another. Using the Oosterwijik prior yields similar Bayes Factors for each study (our study, Domes et al., 2007; Radke and de Bruijn, 2015) (see Table 2 for details). Plots including the posterior- and prior density distribution of the effect sizes are presented in the supplemental material (Figures S1-S6).

**Table 2:** Summary of statistics and descriptive for previous studies

	Current	Radke et al. (2015)	Domes et al. (2007)
	OT vs. PLC	OT vs. PLC	OT vs. PLC
	RMET in %		
*t*-test	72.92 (7.9) vs. 78.06 (7.1) *t*(19) = 2.47, *p* = 0.023 (two-sided), d = 0.55, 95% CI [0.08, 1.02]	68.9 (12.6) vs.67.8 (12.6) *t*(23) = 0.465, *p* = 0.643 (two-sided), d = 0.09, 95% CI [-0.31, 0.49]	72.4 (8.6) vs. 69.4 (8.1) *t*(29) = −2.18, *p* = 0.019 (one-sided), d = 0.40, 95% CI [−0.77, 0.02]
BF10 (BF01)	Informed prior: 6.93 (0.14); Mdn = 0.34; 95% CI [0.13, 0.75] Oosterwijk prior: 9.70 (0.10); Mdn = 0.39; 95% CI [0.18, 0.66]	Informed prior: 0.60 (1.66); Mdn = 0.24; 95% CI [−0.09, 0.44] Oosterwijk prior: 0.534 (1.87); Mdn = 0.29; 95% CI [0.01, 0.48]	Informed prior: 3.29 (0.30); Mdn = −0.30; 95% CI [−0.68, −0.02] Oosterwijk prior: 3.52 (0.28); Mdn = −0.22; 95% CI, [−0.60, −0.01]
	RMET item classification in %		
Easy vs. difficult	81.5 (±8.8) vs. 69.4 (±12.8)	73.6 (±2.5) vs. 63.1 (±2.6)	*N.A.*
Positive vs. negative	78.8 (±11.5) vs. 73.2 (±11.4)	72.8 (±3.0) vs. 63.4 (±2.4)	*N.A.*
High vs. low	80.4 (±8.7) vs. 70.6 (±11.4)	74.3 (±2.2) vs. 62.4 (±2.8)	*N.A.*
Female vs. male	78.4 (±10.0) vs. 72.9 (±10.0)	68.6 (±2.6) vs. 68.0 (±2.6)	*N.A.*
MDMQ			
Arousal	33.9 (±4.7) vs. 34.6 (±3.5)	*N.A.*	34.3 (±4.7) vs. 34.0 (±4.8)
Wakefulness	35.8 (±2.8) vs. 33.9 (±4.3)	*N.A.*	28.8 (±6.8) vs. 28.2 (±7.1)
Mood	30.8 (±3.3) vs. 31.4 (±3.1)	*N.A*.	35.6 (±4.1) vs. 34.2 (±4.5)
	Questionnaires		
EQ	37.2 (±7.6)	33.8 (±10.8)	*N.A.*
IRI-PT	23.1 (±3.1)	22.7 (±5.2)	*N.A.*
IRI-EC	22.6 (±2.0)	21.9 (±2.8)	*N.A.*
IRI-F	22.2 (±4.6)	22.5 (±4.8)	*N.A.*
IRI-PD	18.4 (±3.2)	16.8 (±4.3)	*N.A.*
BIS-total	63.0 (±9.0)	*N.A.*	*N.A.*
BIS-attentional	16.9 (±3.2)	*N.A.*	*N.A.*
BIS-motor	23.1 (±3.7)	*N.A.*	*N.A.*
BIS-nonplanning	23.0 (±4.7)	*N.A.*	*N.A.*

Means with standard deviations except for *t*-test results. Effect size d according to Cohen (Cohen, 1988): small effect *d* = 0.2, medium effect *d* = 0.50, large effect *d* = 0.80. BF_10_: Bayes factor to quantify evidence for the alternative hypothesis relative to the null hypothesis; BF_01_: Bayes factor to quantify evidence for the null hypothesis relative to the alternative hypothesis; Mdn = median effect size and 95% credible intervals of the posterior distribution of the effect size; BIS, Barratt Impulsiveness scale; EQ, empathy quotient; IRI, Interpersonal Reactivity Index; IRI-PT, IRI perspective taking; IRI-EC, IRI empathic concern; IRI-F, IRI fantasy; IRI-PD, IRI personal distress; MDMQ, Multidimensional Mood Questionnaire; N.A., not available; OT, oxytocin; PLC, placebo; RMET, Reading the Mind in the Eyes Test.

### RMET item classification

Repeated measure ANOVAs revealed main effects of condition (OT vs. PLC) and most item classifications (task difficulty, task intensity, sex), except for valence (Table S4, supplemental material). In both conditions, participants achieved higher RMET scores in easier compared with more difficult items (F(1, 137.5) = 19.83, *p* ≤ 0.001, η_*p*_^2^ = 0.51), in items with higher intensity compared to items with lower intensity (F(1, 137.5) = 24.32, *p* ≤ 0.001, η_*p*_^2^ = 0.56), and in items that depicted a female rather than a male eye region (F(1, 135.8) = 8.14, *p* = 0.010, η_*p*_^2^ = 0.30). None of the condition-classification-interactions reached significance (all *p*s > 0.30), suggesting that OT does not affect the recognition of mental states depending on difficulty, valence, intensity, and sex of the presented facial expression. Every Bayesian ANOVA compared five models: (1) the null model, (2) the model with condition (PLC vs. OT), (3) the model with task classification (difficulty, valence, intensity, sex), (4) the model with both main effects (condition, task classification), and (5) the model with both main effects (condition, task classification), and their interaction (Table S5, supplemental material). For the model comparisons, including task difficulty, and intensity, the models, including the two main effects—condition and task classification—provide the strongest support against the null model. Regarding the task valence and the item classification of sex, the models with the main effect of condition provided the strongest support, although the evidence favoring these models was small.

### Mood state

The subdomain Wakefulness was significantly higher in the OT condition, *t*(19) = −2.17, *p* = 0.043, *d* = 0.49, 95% CI [0.02, 0.94], BF_10_ = 0.11, Mdn = −0.44; 95% CI [−0.88, −0.01]. However, according to the Bayes factor it is 8.8 (BF_01_) times more likely that there was no difference in Wakefulness between the conditions. Notably, participants in the current study scored higher in Wakefulness in both the OT and the PLC study visit compared with Domes’ et al. study (OT: *t*(48) = −4.35, *p* < 0.001, *d* = 1.27, 95% CI [−0.64, 1.87], BF_10_ = 2.76, Mdn = −0.99; 95% CI [−1.67, −0.18]), for PLC: *t*(48) = −3.21, *p* = 0.002, *d* = 0.93, 95% CI [0.33, 1.52], BF_10_ = 0.27, Mdn = −0.45; 95% CI [−1.20, 0.30]). Correlational analysis of the suspected association between Wakefulness and the RMET score in the PLC and OT condition did not reveal a significant association in the current study (PLC: *r* = −0.06, *p* = 0.786, 95% CI [−0.39, 0.49], BF_10_ = 0.29; OT: *r* = −0.30, *p* = 0.195, 95% CI [−0.65, 0.16], BF_10_ = 0.61). The MDMQ subdomains Mood and Arousal did not differ significantly between PLC and OT study visits (all *p*s > 0.28, all BFs_10_ > 1.42, see Table 2 for descriptive statistics).

### Trait empathy

**Fig. 3:**
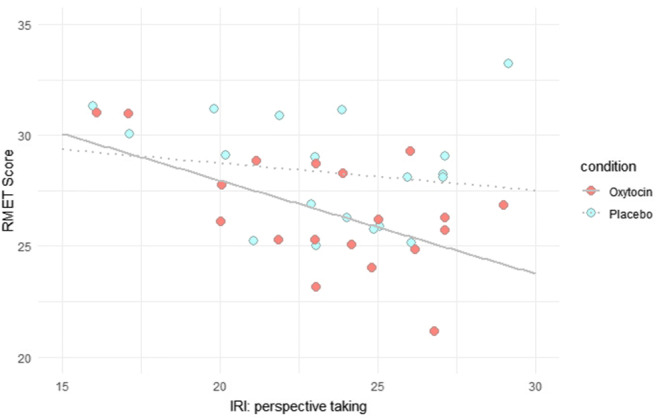
Correlation of the RMET score and perspective taking (IRI-PT). Scatterplot with regression lines for the RMET score in the PLC (red dots, solid line) and the OT (blue dots, dotted line) condition and their relation to the perspective taking scale of the IRI (IRI-PT). In the OT but not the PLC condition, the RMET score was negatively correlated with perspective taking. Note that one participant did not answer the online questionnaires.

None of the online questionnaires measuring trait empathy (EQ, IRI) or impulsiveness (BIS) were related to the RMET score in the PLC condition (all *r*s < |0.33|, all *p*s > 0.17, all BFs_10_ < 0.68 ). In the OT condition, the IRI subdomain Perspective Taking (IRI-PT) correlated negatively with the RMET score (*r* = −0.63, *p* = 0.004, 95% CI [−0.84, −0.24], BF_10_ = 13.41), indicating that subjects with higher self-reported perspective taking abilities achieved a lower RMET score in the OT condition (Figure 3).

This was accompanied by a Bayes factor of 13.41, indicating strong evidence that there exists an association between perspective taking as measured by the IRI and the RMET score in the OT condition. Except for very small prior widths, the robustness check shows that the Bayes factor is relatively stable across different prior widths (supplemental material, Figure S7). When controlling for PLC RMET performance in a partial correlation, the IRI-PT scale remained significantly correlated with the RMET (*r* = −0.63, *p* = 0.004, 95% CI [−0.85, −0.23]). Other scales of the IRI (IRI-EC, IRI-F, IRI-PD), the EQ, and the BIS were not significantly related to RMET performance in the OT condition (all *rs* < |0.40|, all *ps* > 0.09, all BFs_10_ < 1.10).

## Discussion

We investigated the influence of OT on “mind-reading,” as assessed with the RMET, explored its relationship with empathic abilities, and extended our results by including peripheral serum OT measures. Opposed to prior results that reported an improvement of mentalizing abilities (Domes et al., 2007) or null-effects (Radke & de Bruijn, 2015), our findings point toward an impairing effect of OT. Individuals performed significantly worse after administration of intranasal OT compared with PLC. Specifically, we found a Bayes factor of 6.93, meaning that there is 6.93 times more evidence for the alternative hypothesis that OT influences mind-reading. Domes` et al. (2007) study shows that the alternative hypothesis is 3.29 times more likely than the null model providing evidence for a positive effect of OT on “mind-reading,” whereas Radke and de Bruijn’s (2015) study does not favor one hypothesis over another. Furthermore, OT did not affect mentalizing abilities depending on item classification, which is in accordance with Radke and de Bruijn’s (2015) findings and is opposed to other studies (Domes et al., 2007; Feeser et al., 2015). However, RMET item classification influenced participant’s accuracy independently of OT effects as in Radke and Bruijn’s study (2015), showing that participants found it more difficult to label difficult emotions, less intense emotions, and emotions expressed by a male’s eye region. Furthermore, we found no significant change from pre- to post-OT serum levels after intranasal OT administration.

By reporting negative effects of OT on “mind-reading,” this study further reinforces uncertainties regarding OT effects in the field of empathy research. In the following, we discuss several factors that might explain diverging and converging results in the three studies that investigated this question (our study, Domes et al., 2007; Radke and de Bruijn, 2015) and subsequently present more general considerations.

(1) Study populations and sample size: our sample comprised a mixed population compared with student-only populations in the other two studies and our sample size was similarly small. Small sample sizes have been generally criticized in studies that investigate intranasal OT effects (Walum et al., 2016). However, despite the small sample size, we report a medium-effect size (*d* = 0.55), which can be considered quite large compared to the average effect sizes (*d* = 0.28) reported in the OT literature (Walum et al., 2016). Additionally, the finding of this study fits the conclusion of a recent systematic review, which showed that statistical significance is generally unrelated to sample size in intranasal OT research (Mierop et al., 2020).

(2) Nonspecific OT effects: in our study, but not Domes et al. (2007) study, participants reported higher wakefulness in the OT compared to the PLC condition. Wakefulness is associated with the activity of an evolutionarily conserved arousal system that regulates different neural populations and neurotransmitter secretion (de Lecea et al., 2012) and could possibly interact with OT effects. Differences in wakefulness could therefore have led to differential results in the two studies. Nevertheless, we could not confirm a direct link between RMET performance and wakefulness.

(3) Trait empathic abilities: in line with Radke and de Bruijn’s (2015) study, our findings (EQ; IRI) were not related to the RMET score in the PLC condition. However, we found evidence for a negative relationship between Perspective Taking (IRI-PT) and the RMET score after OT administration, suggesting that OT impaired mind-reading in individuals with higher perspective taking skills. A similar negative relation was found in Radke and de Bruijn’s study for the IRI subscale Empathic Concern (IRI-EC). Differences in empathy scores between studies might partially explain contradictory effects of OT on RMET scores in the different studies. It has been reported that individuals with low baseline empathic abilities are more likely to profit from exogenous OT (Feeser et al., 2015). Notably, our participants showed higher self-measured empathy and RMET scores than subjects participating in Radke and de Bruijn’s (2015) study. OT modulates its own release resulting in a non-linear relationship or a saturation curve that could be related to impairing effects of OT in individuals with high empathic abilities. Such impairing effects might reflect a disturbance of homeostatic processes. Furthermore, the finding that the EQ is not related to the RMET performance is consistent with earlier findings (Launay et al., 2015; Radke & de Bruijn, 2015).

(4) Waiting period: Keeping human interaction to a minimum possibly influenced the OT effect in our study, as positive and negative social stimuli likely affect the OT system (Neumann & Landgraf, 2012). This was handled differently in other studies; for example, participants in Radke and de Bruijn’s study (2015) completed questionnaires or could study, read, or engage in other activities, while participants in Domes’ et al. study (2007) could read news magazines.

Recent overarching models of the effects of OT on human behavior might help us to understand the impairing effects of OT on “mind-reading.” First, the strictly nonsocial (i.e., keeping social interaction to a minimum) and even threatening environment (intravenous blood draw) could have negatively influenced the effect of OT. According to the allostatic theory (Quintana & Guastella, 2020), OT facilitates stability in changing environments to promote survival. Our study setting possibly induced an adaptive response to the threat, hereby promoting nonsocial behavior in terms of withdrawal and a reduced ability of mental state recognition. Similarly, this study setting could also explain the negative findings within the general approach-avoidance model, suggesting that OT motivates approach to rewards and reinforces avoidance to threats (Harari-Dahan & Bernstein, 2014). Finally, the social salience hypothesis states that OT modulates the salience of social stimuli depending on interindividual properties, such as empathic abilities, personality traits, degree of psychopathology, or the present psychological state (Shamay-Tsoory & Abu-Akel, 2016). Our sample showed high baseline empathic abilities and the BIS impulsivity score was consistent with results found in healthy males (Stanford et al., 2009). In socially proficient individuals, OT application possibly causes an impairing effect on “mind-reading” by inducing oversensitivity to emotions and thereby impairing their accurate classification. Lastly, we think that further nonspecific effects could have influenced task performance. For instance, participants in our study were not allowed to eat before study onset for a longer time window compared with the other studies. Moreover, they received financial compensation and were more wakeful. Because OT is associated with a broad range of human behaviors, such additional confounding variables could potentially explain these divergent study results.

Beyond that, more general considerations have to be taken into account. Psychological tasks, such as the RMET, might not be an ideal measure to assess the effect of OT on social cognition. Considering that there is context-dependent variability of OT effects (Bartz et al., 2011), mental state recognition of faces presented out of context and without movement or body language might differ entirely from mental state recognition in a real social context. Moreover, the RMET depends on language abilities (Olderbak et al., 2015; Peterson & Miller, 2012) and higher-order executive brain functions (Launay et al., 2015; Mitchell & Phillips, 2015), which again might be related to previously mentioned sample characteristics (see discussion point 1). Studies investigating the effects of OT on other ToM measures also report mixed findings. Woolley et al. (2014) included “The awareness of Social Inference Test,” which is a dynamic emotion recognition task consisting of several short videoclips. The authors found no improvement of emotion recognition after intranasal OT administration. Another study reported impairing effects of OT on emotion recognition using the “Perceiving Emotions” and “Understanding Emotions” tasks of the Mayer-Salovey-Caruso Emotional Intelligence Test. Participants receiving intranasal OT rated facial emotions as more intense than those receiving PLC. This effect was associated with a decrease in accuracy (Cardoso et al., 2014). By contrast, Bartz et al. (2019) found that intranasal OT improves empathic accuracy of participants who rated the valence of feelings of persons discussing emotional autobiographic events. This effect was limited to men scoring higher on autistic traits. In accordance with our study, the authors report slightly worse task performance for socially proficient men after OT administration.

Studies investigating OT are usually limited to male samples to keep hormonal variance low. However, males show differential brain activation patterns compared to females after OT administration (Domes et al., 2007; Tully et al., 2018) and higher levels of testosterone can have an opposing action to OT (Crespi, 2016). In healthy women, the effect of intranasal OT might be entirely different. 

Moreover, it needs to be considered that the effect of OT on RMET performance could simply reflect a regression to the mean. Statistical variation could be mistaken as real change and lead to the incorrect conclusion that OT influences “mind-reading.”

Even though intranasal OT can produce effects in brain physiology, there is limited evidence that it can reliably manipulate behavioral effects (Winterton et al., 2021). Furthermore, it remains unclear whether peripheral OT can be used as a valid and reliable marker of OT circulation. Regarding OT plasma levels, it has been suggested that they are not representatively mirroring levels in the brain even though increased plasma OT levels have been reported after intranasal OT application (Gossen et al., 2012; Striepens et al., 2013).

We found no significant change of OT serum levels after intranasal application, which might be related to methodological issues. Recent publications report that different OT assays lack reliability and yield widely varying OT values (Lefevre et al., 2017; Poljak & Sachdev, 2021). In this study, baseline variability in OT levels was high and mostly exceeded the typical range found in healthy males when applying radioimmunoassay methods. Therefore, we postulate a cautious interpretation on the association of OT serum levels with behavioral measures.

## Conclusions

As opposed to previous results, we found a moderate impairing effect on “mind-reading”-performance after OT administration. Our findings add to the heterogeneity of earlier findings and provide evidence that OT might even interfere with social cognitive abilities. This emphasizes the importance of identifying intra- and interindividual factors that determine OT effects. Specifically, different sample characteristics, including baseline empathic abilities as well as differences in study designs, might explain divergent results regarding OT-effects on RMET-performance. Future studies should investigate individual hormonal, situational, and cognitive factors that determine OT effects on behavioral outcomes. Furthermore, a better understanding of pharmacokinetic dynamics of different pathways into the central nervous system and the relationship between peripheral and central OT levels is key to understand behavioral effects.

## Supplementary Information

ESM 1 (PDF 450 kb)
