# Supplementary material for: Divergent effects of oxytocin on “mind-reading” in healthy males
Source: Cogn Affect Behav Neurosci. 2021 Sep 13;22(1):112–22. doi: 10.3758/s13415-021-00936-3 (PMC8791897; doi:10.3758/s13415-021-00936-3)
Supplement: Supplementary file 1 — (PDF 450 kb) [file 13415_2021_936_MOESM1_ESM.pdf]

**Supplemental material**

Divergent effects of oxytocin on “mind-reading” in healthy males

| **Table S1.**  *Test of Normality (Shapiro-Wilk) for the RMET.* | | |
| --- | --- | --- |
| **Variable** | **W** | ***p*** |
| RMET score ^a^ | 0.967 | 0.699 |
| RMET difficult, PLC | 0.949 | 0.346 |
| RMET difficult, OT | 0.956 | 0.464 |
| RMET easy, PLC | 0.891 | 0.028 * |
| RMET easy, OT | 0.929 | 0.150 |
| RMET negative, PLC | 0.962 | 0.593 |
| RMET negative, OT | 0.931 | 0.160 |
| RMET positive, PLC | 0.892 | 0.029 * |
| RMET positive, OT | 0.946 | 0.312 |
| RMET high intensity, PLC | 0.900 | 0.042 * |
| RMET high intensity, OT | 0.955 | 0.443 |
| RMET low intensity, PLC | 0.946 | 0.331 |
| RMET low intensity, OT | 0.962 | 0.581 |
| RMET male, PLC | 0.956 | 0.472 |
| RMET male, OT | 0.923 | 0.113 |
| RMET female, PLC | 0.923 | 0.112 |
| RMET female, OT | 0.963 | 0.603 |
| *Note.* Significant results suggest a deviation from normality.  ^a^ difference score (pre vs. post OT application) to calculate a paired-samples *t*-test  * Indicates p < .05. | | |

| **Table S2.**  *Test of Normality (Shapiro-Wilk) for psychometric measures.* | | |
| --- | --- | --- |
| **Variable** | **W** | ***p*** |
| MDMQ Arousal  ^a^ | 0.970 | 0.753 |
| MDMQ Wakefulness  ^a^ | 0.910 | 0.063 |
| MDMQ Mood  ^a^ | 0.932 | 0.165 |
| EQ | 0.970 | 0.774 |
| IRI-total | 0.954 | 0.456 |
| IRI-PT | 0.919 | 0.110 |
| IRI-EC | 0.868 | 0.014 * |
| IRI-F | 0.956 | 0.498 |
| IRI-PD | 0.961 | 0.592 |
| BIS-total | 0.968 | 0.734 |
| BIS-attentional | 0.922 | 0.125 |
| BIS-motor | 0.944 | 0.316 |
| BIS-non-planning | 0.965 | 0.667 |
| *Note.* Significant results suggest a deviation from normality.  ^a^ difference score (pre vs. post OT application) to calculate a paired-samples *t*-test  * Indicates p < .05. | | |

| **Table S3.**  *Test of Normality (Shapiro-Wilk) for OT blood levels.* | | |
| --- | --- | --- |
| **Variable** | **W** | ***p*** |
| OT blood level, PLC  ^a^ | 0.918 | 0.092 |
| OT blood level, OT  ^a^ | 0.922 | 0.110 |
| OT blood level, pre measure  ^a^ | 0.958 | 0.505 |
| OT blood level, post measure  ^a^ | 0.944 | 0.283 |
| OT blood level, PL pre | 0.971 | 0.778 |
| OT blood level, PLC post | 0.939 | 0.230 |
| OT blood level, OT pre | 0.932 | 0.169 |
| OT blood level, OT post | 0.903 | 0.048 * |
| OT blood ratio, PLC | 0.717 | <.001 ** |
| OT blood ratio, OT | 0.758 | <.001 ** |
| *Note.* Significant results suggest a deviation from normality.  ^a^ difference score (pre vs. post OT application) to calculate a paired-samples *t*-test  * Indicates p < .05. ** indicates p <.01 | | |

**Adverse events**

The following adverse events were reported: irritation of the nasal mucosa: n=1 (pre and post drug intake); nausea: *n* = 0; headache: *n* = 2 (pre and post drug intake); change in mood (post drug intake): *n* = 6 (1=tired/sleepy; 2=happy; 3=lifted mood; 4=apathetic; 5=calm; 6=tired); allergic dermatitis: *n* = 1 (wrist eczema); other events: *n* = 5 (1=IV irritation; 2=sleepy; 3=sore throat; 4=woozy; 5=tired). Subjects rated only changes in mood and some of the other events (sleepy, woozy) to be associated with effects of drug intake. Nose bleeding occurred before intranasal OT/ PLC administration in one individual.

**Bayesian paired t-test (current study, Domes et al., 2007; Radke & de Bruijn, 2015)**

**Figure S1.** Prior (informed *d* = 0.28, Cauchy scale = 0.1) and Posterior from the study by Domes et al., 2007


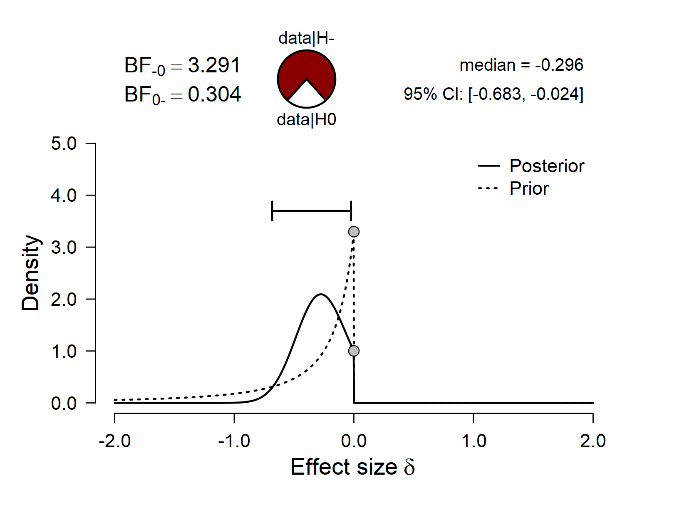


**Figure S2.** Prior (Oosterwijk prior) and Posterior from the study by Domes et al., 2007


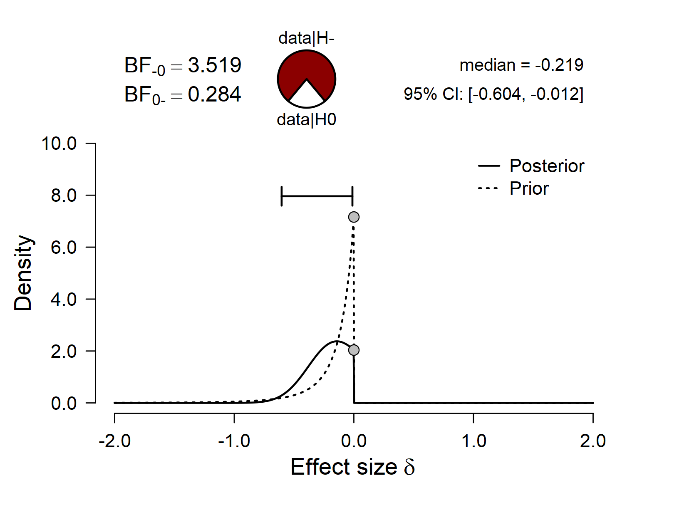


**Figure S3.** Prior (informed *d* = 0.28, Cauchy scale = 0.1) and Posterior from the study by Radke & de Bruijn, 2015


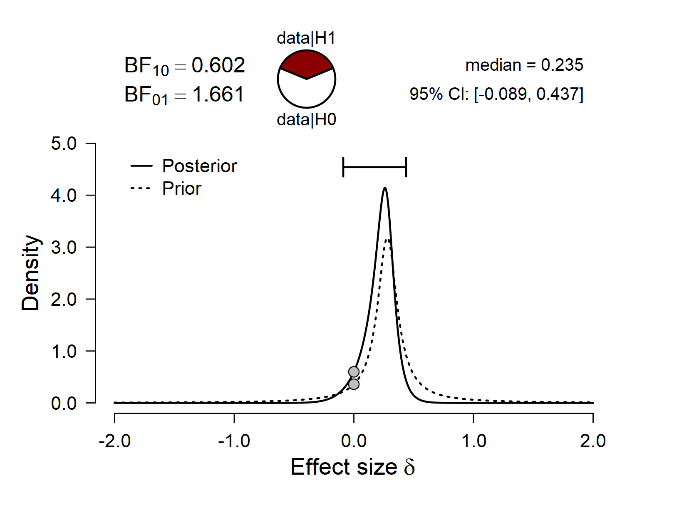


**Figure S4.** Prior (Oosterwijk prior) and Posterior from the study by Radke & de Bruijn, 2015


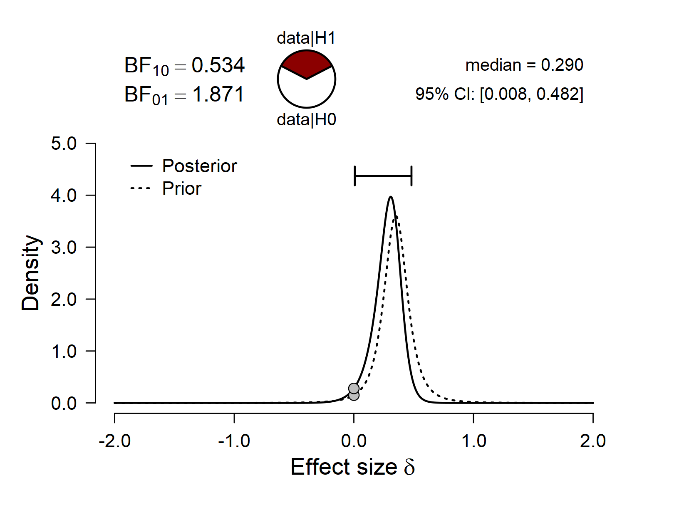


**Figure S5.** Prior (informed *d* = 0.28, Cauchy scale = 0.1) and Posterior from the current study


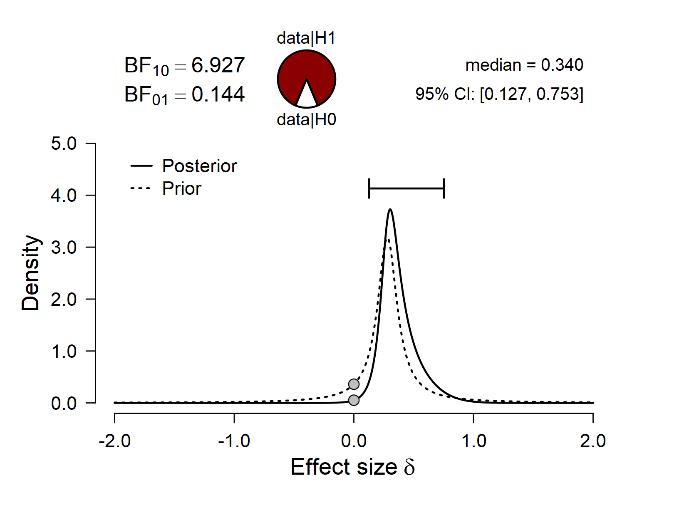


**Figure S6.** Prior (Oosterwijk prior) and Posterior from the current study


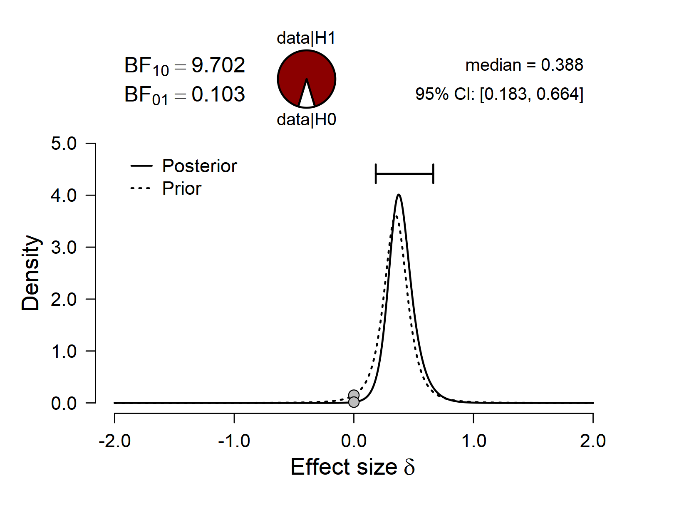


**ANOVA RMET and item classification**

**Table S4**.

*Results of repeated measure ANOVAs for condition (OT or PLC)*

*and respective item classification.*

| Sources | *df* | *F* | Partial η^2^ | *p* |
| --- | --- | --- | --- | --- |
| Condition | 1 | 6.12 | .24 | .023* |
| Task difficulty | 1 | 19.83 | .51 | <.001** |
| Condition x task difficulty | 1 | 0.04 | .00 | .851 |
| Within effects error | 19 | (137.5) |  |  |
| Condition | 1 | 6.42 | .25 | .020* |
| Task valence | 1 | 3.88 | .17 | .064 |
| Condition x task valence | 1 | 0.13 | .01 | .719 |
| Within effects error | 19 | (147.8) |  |  |
| Condition | 1 | 6.12 | .24 | .023* |
| Task intensity | 1 | 24.32 | .56 | <.001** |
| Condition x task intensity | 1 | 0.00 | .00 | 0.949 |
| Within effects error | 19 | (137.5) |  |  |
| Condition | 1 | 6.41 | .25 | .020* |
| Sex | 1 | 8.14 | .30 | .010* |
| Condition x sex | 1 | 1.08 | .05 | .311 |
| Within effects error | 19 | (135.8) |  |  |
| *Note*. Values reported in parentheses are mean-square error values.  * p < .05 ** p < .01 | | | | |

**Table S5.**

*Results of Bayesian repeated measure ANOVAs comparing models with*

*condition, task classification, condition + task classification, and their*

*interaction against the null model.*

| Model type | *P (M\|data)* | BF_M_ | BF_10_ |
| --- | --- | --- | --- |
| Null model | 1.998 e-5 | 7.992e-5 | 1.000 |
| Difficulty | 0.252 | 1.346 | 12600.855 |
| Condition | 2.467e-5 | 9.868e-5 | 1.235 |
| Condition + difficulty | 0.560 | 5.086 | 28015.132 |
| Condition + difficulty + condition*difficulty | 0.188 | 0.929 | 9431.577 |
| Null model | 0.150 | 0.707 | 1.000 |
| Valence | 0.111 | 0.501 | 0.741 |
| Condition | 0.362 | 2.270 | 2.409 |
| Condition + valence | 0.285 | 1.592 | 1.849 |
| Condition + valence + condition*valence | 0.092 | 0.404 | 0.610 |
| Null model | 8.579e-5 | 3.432e-4 | 1.000 |
| Intensity | 0.161 | 0.770 | 1881.682 |
| Condition | 1.720e-4 | 6.881e-4 | 2.005 |
| Condition + intensity | 0.636 | 6.991 | 7414.336 |
| Condition + intensity + condition*intensity | 0.202 | 1.014 | 2357.746 |
| Null model | 0.107 | 0.478 | 1.000 |
| Sex | 0.059 | 0.253 | 0.556 |
| Condition | 0.452 | 3.300 | 4.232 |
| Condition + sex | 0.267 | 1.454 | 2.495 |
| Condition + sex + condition*sex | 0.115 | 0.521 | 1.078 |
| *Note.* $\mathrm{BF}_{10}$BF*_10_* = Bayes factor in favor of the alternative model; P(M\|data) = updated probabilities after having observed the data; BF_M_= degree to which the data have changed the prior model odds. | | | |

##### **Figure S7.** Bayes Factor Robustness Check correlation IRI perspective taking and RMET in the OT condition


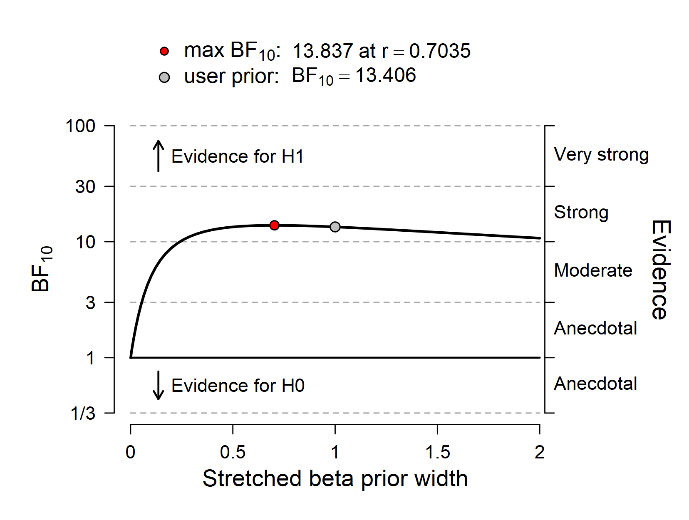


**OT serum blood level analysis**

Given the recent criticism regarding the analysis of peripheral OT quantifications, we recommend caution in the interpretation of the following results.

Descriptive statistics showed an increase in OT blood levels from baseline to post in the OT condition (OT pre: M = 13.40 pg/ml, SD = 6.68 vs. OT post: M = 16.69 pg/ml, SD = 10.02), while OT blood levels decreased in the PLC condition (PLC pre: M = 14.21 pg/ml, SD = 5.49 vs. PLC post: M = 11.78 pg/ml, SD = 6.12). OT blood levels showed high dispersion. In the OT baseline condition, OT ranged from 4.7-26.3 pg/ml; in the PLC baseline condition, OT ranged from 3.7-24.7 pg/ml. Post blood measures varied from 6.1-41.4 pg/ ml in the OT condition and from 3.7-24.9 pg/ ml in the PLC condition.

In both conditions, OT levels did not change significantly from pre to post (OT: t(19) = -1.46, p = .161, d = 0.33, 95% CI [-.13, .77], BF10 = 0.11, Mdn = 0.12, 95% CI [-0.42, 0.39]; PLC: t(19) = 1.61, p = .125, d = 0.36, 95% CI [-.10, .81], BF10 = 2.96, Mdn = 0.35, 95% CI [0.13, 0.58]). A paired-samples t-test to compare the PLC and the OT pre blood measures showed no significant difference (t(19) = -0.50, p = .623, d = 0.11, 95% CI [-.33, .55], BF10 = 0.63, Mdn = 0.30, 95% CI [0.02, 0.50]), whereas there was a significant difference in post serum OT measures between the conditions (t(19) = -2.33, p = .031, d = 0.52, 95% CI [0.05, 0.98], BF10 = 0.12, Mdn = -0.10, 95% CI [-0.70, 0.34]). However, a difference in OT serum measures was not supported by the Bayes factor. Further correlational analysis including OT serum blood measures are presented in the supplemental material.

Additional correlational analysis was applied to investigate associations between serum OT levels and behavioral effects. Due to normality deviations (see Table S3), we applied non-parametric methods. For OT serum measures (Bayesian) Kendall’s correlation coefficient was applied. To determine if OT change depends on the baseline OT level it was correlated with the pre OT level in either condition. In order to assess reliability of blood measures, OT serum levels at baseline were correlated with OT serum levels at 45 min after intranasal administration of PLC and its related baseline measures one week apart. Furthermore, correlations between baseline OT serum measures, OT ratios^a^ and psychometric measures (IRI, EQ, BIS) were investigated.

There was a negative correlation of pre OT blood levels and OT ratios^a^ in the OT but not the PLC condition (PLC: τ = -.17, *p* = .299, BF_10_ = 0.48; OT: τ = -.35, *p* = .034, BF_10_ = 2.48), indicating that in the OT condition higher baseline OT was associated with a smaller OT change in serum levels. The Bayes factor was not robust across different prior widths (see **Figure S8**) and only indicated anecdotal evidence for this association. There was no meaningful association between pre and post blood measures of OT in the PLC condition (τ = .25, *p* = .126, $\mathrm{BF}_{10}$ BF_10_ = 0.88) and the pre blood measures in the OT condition (τ = .19, *p* = .242, $\mathrm{BF}_{10}$ BF_10_ = 0.55). We neither found an association of RMET performance with OT ratios^a^ (PLC: τ = .00, *p* = 1.00, $\mathrm{BF}_{10}$ BF_10_ = 0.29; OT: τ = .06, *p* = .742, $\mathrm{BF}_{10}$ BF_10_ = 0.30) nor OT post blood measures (PLC: τ <.01, *p* = .974, $\mathrm{BF}_{10}$ BF_10_ = 0.29; OT: τ = .03, *p* = .869, $\mathrm{BF}_{10}$BF_10_ = 0.29).

Empathy measures were unrelated to baseline blood measures (Empathy: all *rs* < |.25|, all *ps* > .31, all BFs < 0.79), whereas the BIS Impulsivity score was positively associated with baseline blood measures in either condition (PLC: τ =.39, *p* = .021,$\mathrm{BF}_{10}$ BF_10_ = 3.85; OT: τ = .38, *p* = .025, $\mathrm{BF}_{10}$BF_10_ = 3.21). The Bayes Factor supported this association but for wider prior distributions there is a small drop in the Bayes factor (see **Figure S8, Figure S9**). None of the second order factors of the BIS Impulsivity scale were significantly associated with the OT baseline blood measures (all τs <|.32|, all *ps* > .05, all$\mathrm{BF}_{10}$ BFs_10_ > 1.72). In the PLC and OT condition empathy measures (IRI, EQ) were unrelated to OT ratios (all τs <|.24|, all ps > .16, all BFs10 > 0.74).

^a^ A ratio between pooled pre (first, second and third blood measure) and post serum measures (fourth blood measure drawn 45 min later) was calculated as an indicator of OT uptake into systemic circulation after intranasal application.

##### **Figure S8.** Bayes Factor Robustness Check correlation pre OT blood level and OT blood ratio ^a^ in the OT condition


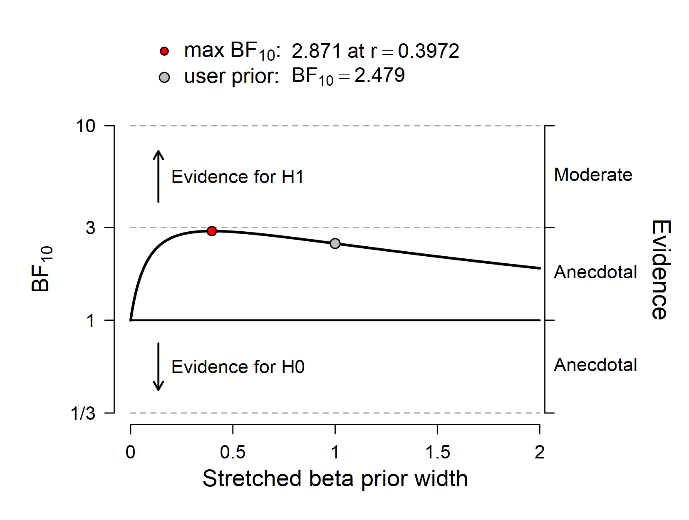


**
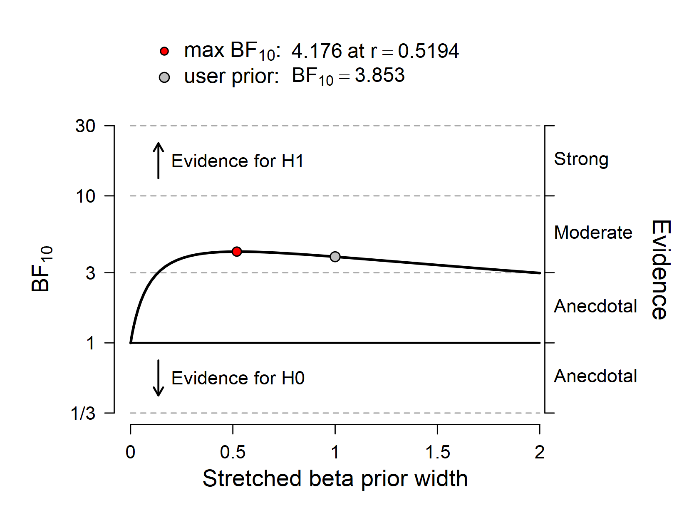
Figure S9.** Bayes Factor Robustness Check correlation BIS impulsivity score and OT pre blood level in the PLC condition


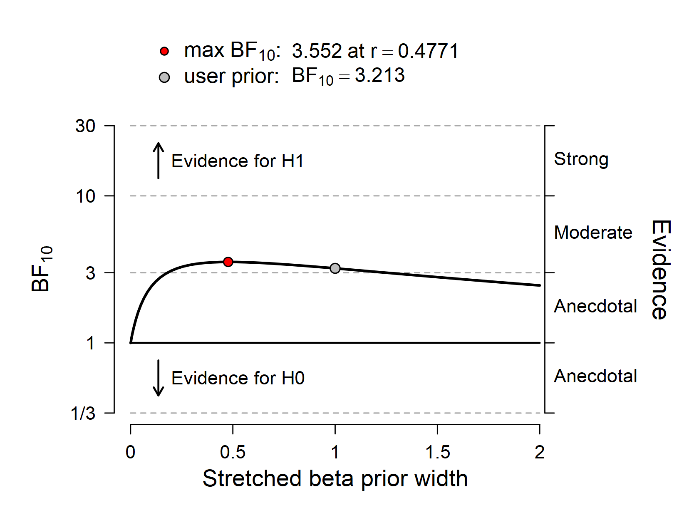
**Figure S9.** Bayes Factor Robustness Check correlation BIS impulsivity score and OT pre blood level in the OT condition

**Normality deviations**

RMET item classification: A few of the inner subject factors of the RMET did not follow a normal distribution (see Table S1). Data transformation as applied with the “bestNormalize” Package in R (Peterson & Peterson, 2020) could not improve normality. However, the repeated measure ANOVA is assumed to be relatively robust to normality violations (Berkovits et al., 2000).

Trait empathy: The subscale IRI-EC was not normally distributed (see Table S2). Additional correlations using Kendall’s tau were applied. These were concordant with parametric methods and did not show a relationship between the IRI subdomain Empathy Concern (IRI-EC) and the RMET (PLC: τ = 0.23, p = .204, 95% CI [-.10, -.52], BF10 = ; OT: τ = −.09, p = .611, 95% CI [-.40, .24], BF10 = ).

**References**

Berkovits, I., Hancock, G. R., & Nevitt, J. (2000). Bootstrap resampling approaches for repeated measure designs: Relative robustness to sphericity and normality violations. *Educational and Psychological Measurement*, *60*(6), 877–892.

Domes, G., Heinrichs, M., Michel, A., Berger, C., & Herpertz, S. C. (2007). Oxytocin improves „Mind-Reading“ in humans. *Biological Psychiatry*, *61*(6), 731–733. https://doi.org/10.1016/j.biopsych.2006.07.015

Peterson, R. A., & Peterson, M. R. A. (2020). Package ‘bestNormalize’. *Published online*, *27*.

Radke, S., & de Bruijn, E. R. A. (2015). Does oxytocin affect mind-reading? A replication study. *Psychoneuroendocrinology*, *60*, 75–81. https://doi.org/10.1016/j.psyneuen.2015.06.006
